# Dexmedetomidine ameliorates diabetic cardiomyopathy by inhibiting ferroptosis through the Nrf2/GPX4 pathway

**DOI:** 10.1186/s13019-023-02300-7

**Published:** 2023-07-10

**Authors:** Fan Li, Zhenfei Hu, Yidan Huang, Haiting Zhan

**Affiliations:** Department of Anesthesiology, First Afiliated Hospital of Xinjiang Medical University, Xinjiang Perioperative Organ Protection Laboratory (XJDX1411), No.1 Liyushan Road, Urumqi, Xinjiang 830054 China

**Keywords:** Diabetic cardiomyopathy, Dexmedetomidine, Ferroptosis, Nrf2/GPX4 pathway, Oxidative stress, H9C2 cells

## Abstract

**Objective:**

Dexmedetomidine (DEX) has been shown to have anti-apoptotic effects in diabetes mellitus, but its role in mitigating diabetic cardiomyopathy (DCM) through ferroptosis regulation is unclear.

**Methods:**

An in vitro DCM model was established using H9C2 cells induced with high glucose (HG) and treated with DEX at varying doses and a nuclear factor erythroid 2-realated factor 2 (Nrf2) specific inhibitor ML385. Cell viability was evaluated using the MTT method after treatment with DEX or mannitol (MAN), and the dosage of DEX used in subsequent experimentation was determined. The effects of HG-induced high osmotic pressure were assessed using MAN as a control. Cell apoptosis was evaluated using flow cytometry. Protein levels of Bcl2, Bax, nuclear Nrf2, and glutathione peroxidase 4 (GPX4) were measured using Western blot. Superoxide dismutase (SOD) activity, malondialdehyde (MDA) levels, Fe^2+^ concentration and reactive oxygen species (ROS) levels were measured using corresponding kits and dichlorodihydrofluorescein diacetate, respectively.

**Results:**

Treatment with DEX or MAN had no effect on H9C2 cell viability. HG induction reduced H9C2 cell viability, increased cell apoptosis, upregulated levels of Bax, Fe^2+^, MDA, and ROS, and downregulated Bcl2 protein levels, SOD activity, and protein levels of nuclear Nrf2 and GPX4. DEX inhibited HG-induced H9C2 cell apoptosis, promoted Nrf2 nuclear translocation, and activated the Nrf2/GPX4 pathway. Inhibition of Nrf2 partially reversed the protective effects of DEX against HG-evoked H9C2 cell injury.

**Conclusion:**

Our findings demonstrate that DEX attenuates HG-induced cardiomyocyte injury by inhibiting ferroptosis through the Nrf2/GPX4 pathway, providing potential therapeutic targets for DCM treatment.

## Introduction

The surging prevalence of diabetes mellitus and heart failure represents a significant public health burden worldwide, and both conditions are independent risk factors for one another [1]. Diabetic cardiomyopathy (DCM) is a pathophysiological abnormality of cardiac structure and function in diabetic patients without hypertension, coronary artery disease, and other types of heart diseases [2]. The pathogenesis of DCM involves a complex interplay of systemic metabolic disorders, subcellular component abnormalities, oxidative stress, inflammation, dysfunctional immune modulation, and inappropriate activation of the renin-angiotensin-aldosterone system [3]. Ferroptosis, a novel form of iron-dependent cell death, has emerged as a potential contributor to the development of DCM, given its critical involvement in the pathological processes of iron accumulation and lipid peroxidation [4, 5]. However, the precise molecular mechanisms underlying the role of ferroptosis in DCM remain poorly understood and warrant further investigation.

Dexmedetomidine (DEX), a highly selective α2 adrenergic agonist primarily used for sedation [6], possesses an array of diverse pharmacological properties, including cardiac protection, anti-inflammatory, sedative, anesthesia, sleep-enhancing, bowel recovery, and sore throat-relieving effects [7]. In rats with diabetes, DEX has been shown to alleviate cardiac dysfunction and improve autophagic dysfunction, indicating its potential anti-autophagic effects in DCM patients [8]. Furthermore, DEX has been demonstrated to exert cardioprotective effects by inhibiting ferroptosis in cardiac ischemia/reperfusion injury [9]. Despite these promising findings, it remains unclear whether DEX can attenuate DCM by suppressing ferroptosis in cardiomyocytes, and this question remains an active area of investigation.

Nuclear factor erythroid 2-related factor 2 (Nrf2) serves as a regulator of cellular antioxidant responses, lipid peroxidation, and ferroptosis [10]. Nrf2 demonstrates its protective function by translocating into the nucleus to counteract organ dysfunction [11]. Additionally, glutathione peroxidase 4 (GPX4) is a crucial regulator of ferroptosis and can be transcriptionally regulated by Nrf2 [12]. DEX has been shown to exert myocardial protection by activating the Nrf2/heme oxygenase-1/solute carrier family 7 member 11/GPX4 axis [13]. In contrast, protein arginine methyltransferase 4 inhibits the Nrf2/GPX4 pathway, thereby accelerating ferroptosis in doxorubicin-induced cardiomyopathy [14]. Furthermore, curcumin has been found to mitigate glucose-induced ferroptosis of cardiomyocytes by facilitating Nrf2 nuclear translocation and reducing excessive GPX4 loss [15]. However, the precise mechanism by which DEX attenuates high glucose (HG)-induced cardiomyocyte ferroptosis by activating the Nrf2/GPX4 pathway requires further investigation. Therefore, the primary objective of this study is to explore whether DEX can enhance the nuclear translocation of Nrf2 and upregulate GPX4 to repress ferroptosis and alleviate HG-induced cardiomyocyte injury.

## Materials and methods

### Cell culture and treatment

Rat cardiomyocytes H9C2 cells (The Cell Bank of Type Culture Collection of The Chinese Academy of Sciences, Shanghai, China) were cultured overnight in Dulbecco’s modified Eagle’s medium (DMEM, Hyclone, Logan, UT, USA) containing 1% penicillin-streptomycin (Hyclone) and 10% fetal bovine serum (FBS, Hyclone) at 37^o^C with in humidified 95% air and 5% CO_2_.

H9C2 cells were divided into the following six groups: control (Con) group (treated with 5.5 mmol/L glucose), mannitol (MAN) group (treated with 5.5 mmol/L MAN to exclude the effect of osmotic pressure [16]), DEX group (treated with 5.5 mmol/L glucose + 0.1, 1, 5, 10, and 20 µM DEX for 72 h), HG group (treated with 30 mmol/L glucose), HG + DEX group (treated with 30 mmol/L glucose + 0.1, 1, 5, 10, and 20 µM DEX for 72 h [17]), HG + DEX + ML385 group (treated with 30 mmol/L glucose + 10 µM DEX + 20 µM ML385 for 72 h [17]). ML385 is a specific Nrf2 inhibitor. MAN (SM8120), DEX (YZ-1,179,333), and ML385 (IM1020) were purchased from Solarbio (Beijing, China). DMEMs with normal glucose and HG were obtained from Hyclone. Cells were harvested after 72 h of treatment.

### Cell viability detection

Cell viability of H9C2 cells was measured using the 3-(4, 5-dimethylthiazol-2-yl)-2, 5-diphenyl tetrazolium bromide (MTT) assay. H9C2 cells were seeded in 96-well plates (1 × 10^4^ cells/well) and cultured for 24 h in normal glucose DMEM supplemented with 10% FBS. Next, 0.5 mg/mL MTT solution was added and incubated for 4 h of treatment at 37^o^C, followed by the addition of 100 µL dimethyl sulfoxide solution to each well. A microplate reader (Thermo Fisher Scientific, Rockford, IL, USA) was used to measure absorbance at 490 nm. Cell viability was defined as the optical density ratio of the sample to the control.

### Cell apoptosis detection

H9C2 cells were collected and stained with Annexin V-FITC and propidium iodide (Beyotime, Shanghai, China) for 30 min at room temperature, followed by two rinses with phosphate buffer saline. The CellQuest software (BD Biosciences, Franklin Lakes, NJ, USA) was used to analyze the fluorescence data.

### Measurement of SOD activity and MDA level

The activity of total superoxide dismutase (SOD) and the level of malondialdehyde (MDA) in H9C2 cells were determined using the Total SOD Activity Assay kit (S0109, Beyotime) and MDA Assay kit (S0131, Beyotime), respectively, in strict compliance with the manufacturer’s specifications.

### Measurement of ferrous iron (Fe^2+^) concentration

To determine the levels of Fe^2+^ in H9C2 cells, the iron assay kit (MAK025, Sigma-Aldrich, St. Louis, MO, USA) was utilized following the manufacturer’s instructions.

### Measurement of reactive oxygen species (ROS) level

To determine the levels of ROS in H9C2 cells, dichlorodihydrofluorescein diacetate (DCFH-DA) staining (10 µmol/L at 37 °C) was performed for 20 min according to the instructions of the ROS assay kit (S0033M, Beyotime). Data quantification was carried out using Image J (NIH, Bethesda, MD, USA).

### Western blot

Protein extraction was performed by lysing H9C2 cells using a lysis buffer (Beyotime) containing protease inhibitor cocktails. Protein extraction and concentration determination were performed using the nuclear protein extraction and BCA kits (Beyotime), respectively. The cell lysates were mixed with 5 × Gel Sample Loading Buffer (New Cell & Molecular Biotech, Suzhou, China) and heated at 100 °C for 8 min to denature the proteins. Next, the proteins was then separated by 10% SDS-PAGE and transferred to a PVDF membrane (Merck Millipore, Billerica, MA, USA). After blocking with 5% bovine serum albumin in TBS-Tween, the membrane was incubated overnight at 4 °C with primary antibodies, washed with TBS-Tween, and probed with horseradish peroxidase-conjugated goat anti-rabbit antibody (1/2000, ab6721, Abcam, Cambridge, MA, USA). The protein bands were visualized using ECL detection reagents (Thermo Fisher Scientific), and the signal intensities were quantified using Image J (NIH). The primary antibodies used were anti-Bcl2 (1/1000, ab196495, Abcam), anti-Bax (1/1000, ab32503, Abcam), anti-Nrf2 (1/500, ab62352, Abcam), anti-GPX4 (1/1000, ab125066, Abcam), anti-β-actin (cytoplasmic protein control; 1/1000, ab8227, Abcam) and anti-Lamin A/C (nuclear protein control; 1/10,000, ab133256, Abcam).

### Statistical analysis

Data analysis and graph plotting were performed using GraphPad Prism 8.01 (GraphPad Software, San Diego, CA, USA). Measurement data were presented as mean ± standard deviation. The independent sample *t* test and one-way analysis of variance with Tukey’s test were used for group comparisons. Two-sided tests were used to obtain *p* values, and *p* < 0.05 was considered statistically significant.

## Results

### DEX suppresses HG-induced cardiomyocyte apoptosis

**Fig. 1:**
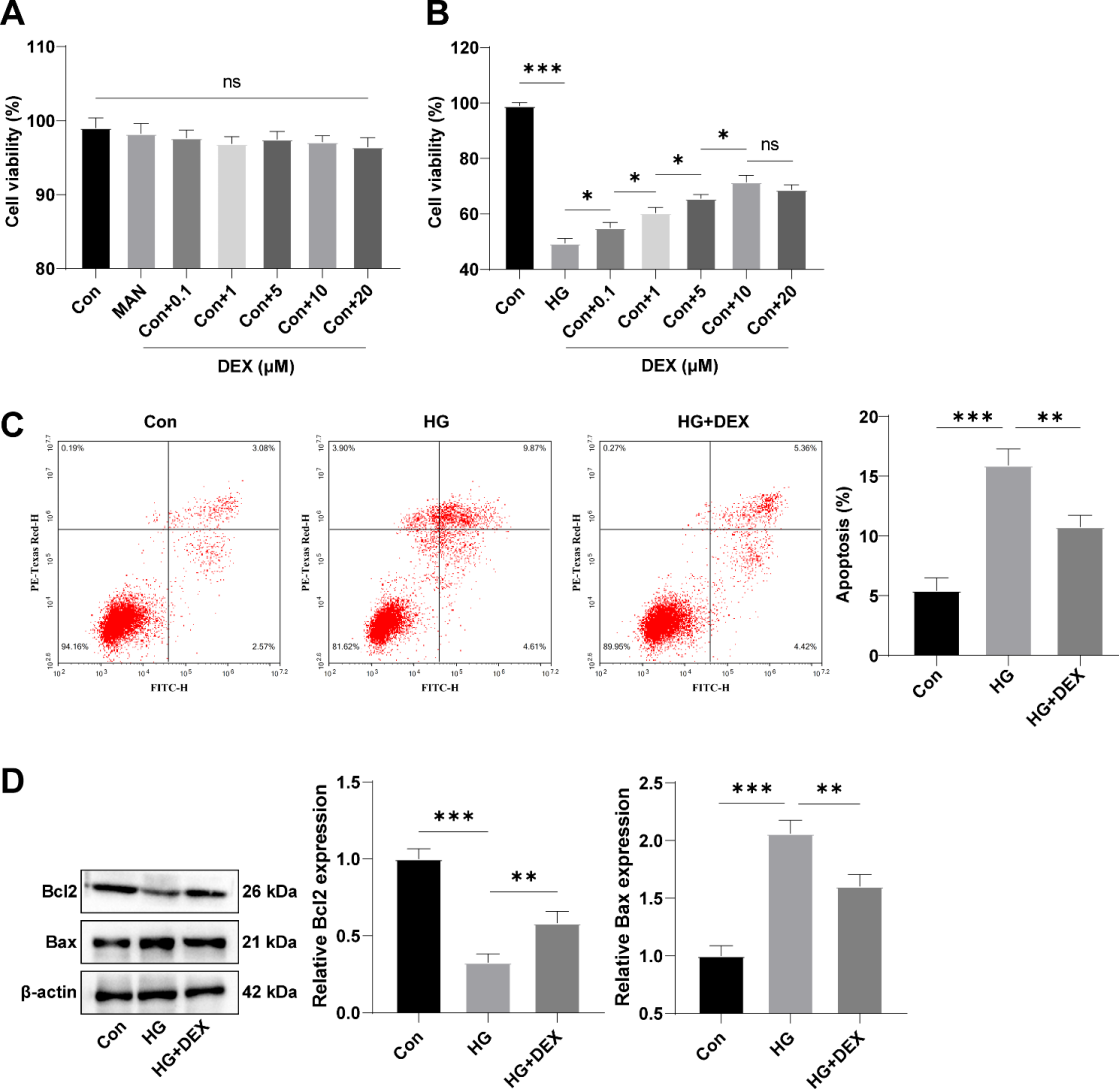
DEX suppresses HG-induced H9C2 cell apoptosis. **(A/B)** Cell viability was detected using the MTT method; **(C)** Cell apoptotic rate was estimated by flow cytometry; **(D)** The protein levels of Bcl2 and Bax were determined by Western blot. Three replicates were guaranteed in cell experiments. Mean ± standard deviation was introduced to represent data. The comparison among multiple groups was made using one-way analysis of variance with Tukey’s test. ns *p* > 0.05, * *p* < 0.05, ** *p* < 0.01, *** *p* < 0.001

H9C2 cells were exposed to either normal glucose or HG and treated with different concentrations of DEX (0.1, 1, 5, 10, and 20 µM). The effect of high osmotic pressure on cells was excluded using MAN treatment as a control. DEX or MAN alone did not affect H9C2 cell viability under normal glucose conditions (Fig. 1A, all *p* > 0.05). However, HG-induced cells showed reduced viability compared to the control group (Fig. 1B, p < 0.001). Treatment with medium and low dose levels of DEX (0.1–10 µM) significantly increased cell viability in a dose-dependent manner, (Fig. 1B, all *p* < 0.05), while 20 µM DEX showed only a slight reduction in cell viability compared to 10 µM DEX, and the difference was not significant (Fig. 1B, p > 0.05). Thus, dosage of 10 µM DEX was used in subsequent experiments. Flow cytometry and Western blot analysis showed that HG-induced cells had a higher cell apoptotic rate and Bax protein levels, and lower Bcl2 protein levels relative to the control group (Fig. 1-C, all *p* < 0.001). Treatment with DEX reduced the apoptotic rate and increased Bcl2 protein levels while decreasing Bax protein levels in HG-induced cells (Fig. 1C-D, all *p* < 0.01). These findings suggest that DEX can suppress HG-induced H9C2 cell apoptosis.

### DEX ameliorates HG-induced oxidative stress and ferroptosis of cardiomyocytes

**Fig. 2:**
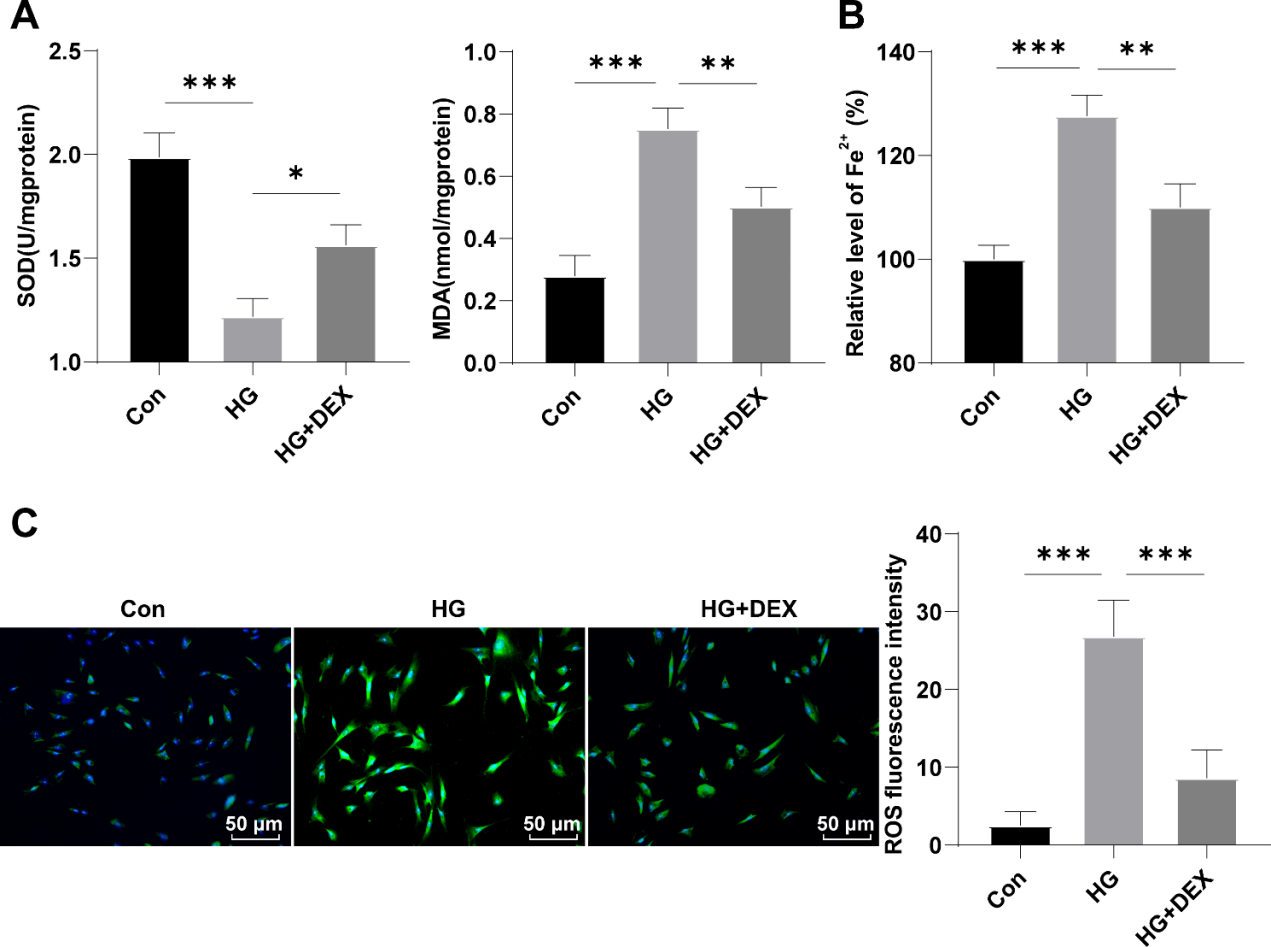
DEX ameliorates HG-induced oxidative stress and ferroptosis of H9C2 cells. **(A)** Total SOD activity and MDA levels; **(B)** Fe^2+^ concentration; **(C)** ROS levels assessed by DCFH-DA. Three replicates were guaranteed in cell experiments. Mean ± standard deviation was introduced to represent data. The comparison among multiple groups was made using one-way analysis of variance with Tukey’s test. * *p* < 0.05, ** *p* < 0.01, *** *p* < 0.001

Subsequently, the effects of DEX on HG-induced oxidative stress and ferroptosis of cardiomyocytes were investigated. H9C2 cells were exposed to HG and treated with different concentrations of DEX. Results showed that HG induction led to a significant reduction in SOD activity and a significant increase in MDA level and Fe^2+^ concentration compared to the control group (Fig. 2A-B, all *p* < 0.001). However, treatment with DEX resulted in a dose-dependent increase in SOD activity and a reduction in MDA level and Fe^2+^ concentration in HG-induced H9C2 cells (Fig. 2A-B, all *p* < 0.05). Furthermore, the use of DCFH-DA demonstrated that ROS levels increased after HG induction, but decreased after DEX treatment (Fig. 2C, p < 0.001). These findings indicate that DEX ameliorates oxidative stress and ferroptosis of HG-induced H9C2 cells.

### DEX potentiates Nrf2 nuclear translocation and activates the Nrf2/GPX4 pathway

**Fig. 3:**
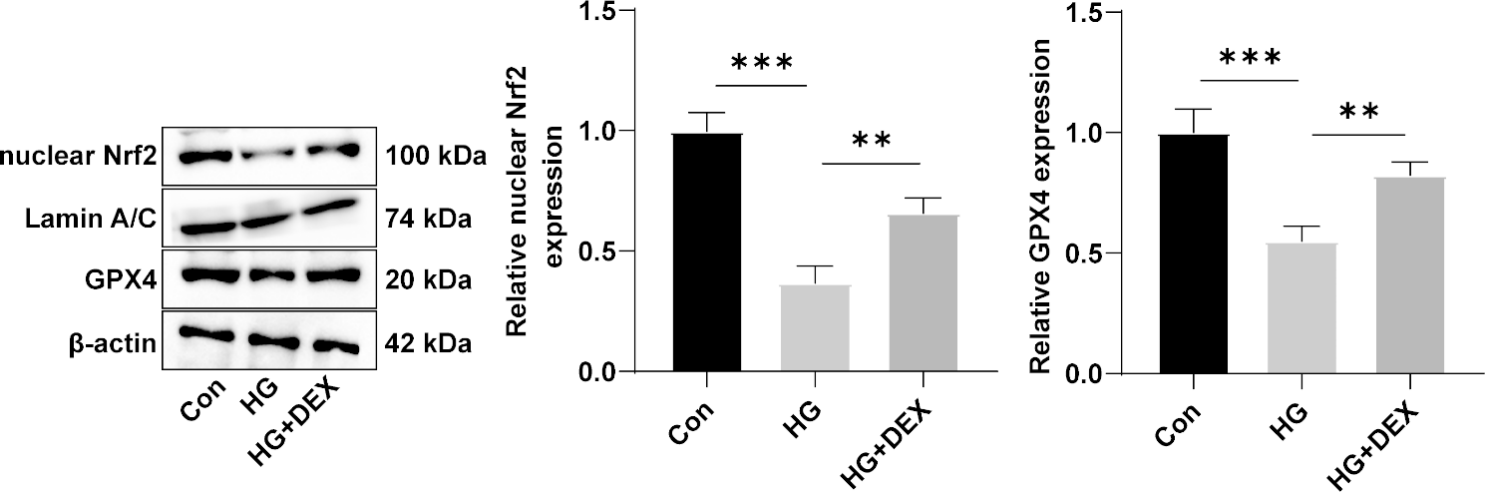
DEX potentiates Nrf2 nuclear translocation and activates the Nrf2/GPX4 pathway. Nuclear Nrf2 expression and GPX4 expression were determined by Western blot. Three replicates were guaranteed in cell experiments. Mean ± standard deviation was introduced to represent data. The comparison among multiple groups was made using one-way analysis of variance with Tukey’s test. ** *p* < 0.01, *** *p* < 0.001

The effects of DEX on Nrf2 nuclear translocation and the Nrf2/GPX4 pathway were investigated. Western blot analysis revealed that HG induction led to a downregulation of nuclear Nrf2 expression and GPX4 expression in H9C2 cells (Fig. 3, all *p* < 0.001). In contrast, the HG + DEX group exhibited higher expression levels of both nuclear Nrf2 and GPX4 compared to the HG group (Fig. 3, all *p* < 0.01). These findings suggest that DEX promotes the nuclear translocation of Nrf2 and activates the Nrf2/GPX4 pathway.

### Nrf2 inhibition reduces the protective function of DEX on HG-induced cardiomyocyte injury

**Fig. 4:**
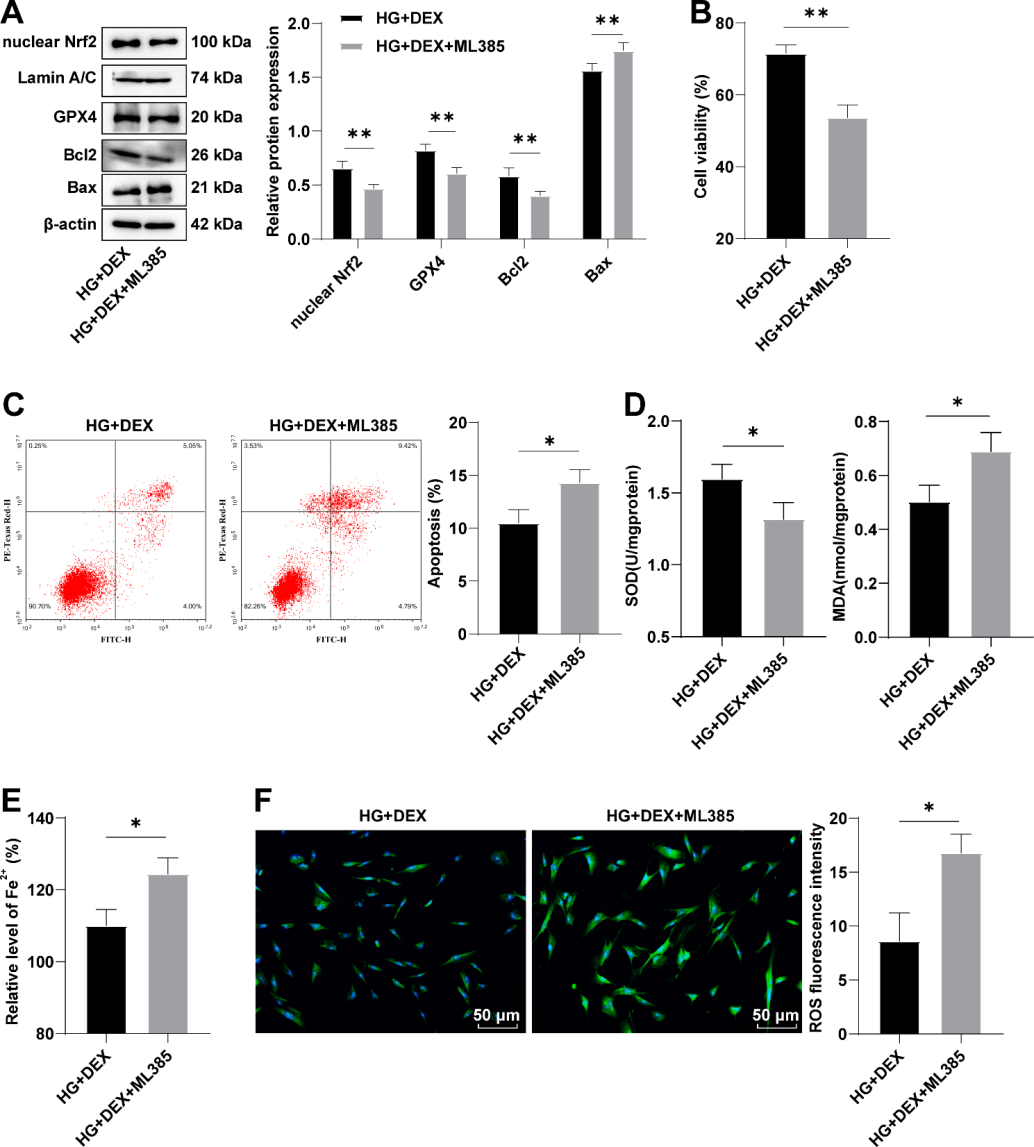
Nrf2 inhibition partially invalidates protective function of DEX on HG-induced cardiomyocyte injury. **(A)** The protein levels of nuclear Nrf2, GPX4, Bcl2 and Bax were determined by Western blot; **(B)** Cell viability was detected using the MTT method; **(C)** Cell apoptotic rate was estimated by flow cytometry; **(D)** Total SOD activity and MDA levels; **(E)** Fe^2+^ concentration; **(F)** DCFH-DA was used to assess ROS levels. Three replicates were guaranteed in cell experiments. Mean ± standard deviation was introduced to represent data. The comparison between 2 groups was made using the independent sample *t* test. * *p* < 0.05, ** *p* < 0.01

To investigate the role of Nrf2 in the protective effect of DEX against HG-induced cardiomyocyte injury, H9C2 cells were treated with both DEX and ML385, a specific Nrf2 inhibitor. The HG + DEX + ML385 group showed lower nuclear Nrf2 levels and GPX4 levels compared to the HG + DEX group (Fig. 4A, all *p* < 0.01), indicating successful downregulation of Nrf2 and GPX4. Furthermore, the HG + DEX + ML385 group exhibited decreased cell viability, increased cell apoptotic rate, downregulated Bcl2 protein and SOD activity, and upregulated Bax protein, Fe^2+^ concentration and levels of MDA and ROS compared to the HG + DEX group (Fig. 4A-F, all *p* < 0.05). These findings suggest that Nrf2 inhibition partially reverses the protective action of DEX against HG-induced H9C2 cell injury.

## Discussion

Despite the identification of numerous targets for the prevention and treatment of DCM, few therapeutic strategies have demonstrated efficacy [18]. Targeting ferroptosis may offer a promising approach to prevent cardiomyopathy [19], as ferroptosis has been shown to contribute to the pathogenesis of DCM and interfering with its pathways may represent a promising strategy to reduce reducing cardiovascular injury [20, 21]. DEX has shown potential as a therapeutic agent for targeting septic heart injury and myocardial ischemia/reperfusion (I/R) injury by modulating ferroptosis [22, 23]. The present study aimed to investigate the therapeutic effects of DEX on HG-induced cardiomyocyte ferroptosis and injury in vitro.

Increased cardiac apoptosis is a major risk factor for for the development of DCM [24]. Consistent with the first set of results presented in this study, HG induction led to a decline in cell viability in cardiomyocytes, while medium and low doses of DEX resulted in enhanced cardiomyocyte viability. The regulation of apoptosis is complex and involves opposing activities of Bcl2 proteins, with apoptosis occurring when Bax and Bak outnumber Bcl2 activities [25]. In this study, DEX treatment resulted in a significant decline in Bax protein expression and an increase in Bcl2 protein expression in HG-induced cardiomyocytes. This is in agreement with previous studies that have documented a reduction in cardiomyocyte apoptosis following DEX treatment in the context of myocardial infarction, as evidenced by an increase in the Bcl-2/Bax ratio [26], as well as in I/R-induced cardiomyocyte injury [27]. These findings suggest that DEX may have a protective effect against HG-induced cardiomyocyte apoptosis.

Mounting evidence suggests that ferroptosis, an iron and ROS-dependent form of cell death, is closely associated with the occurrence and progression of DCM [28–31]. Ferroptosis, mediated by oxidative stress, is critical to the pathogenesis of several cardiovascular diseases [32]. Additionally, oxidative stress is one of the most typical pathogenic characteristics of DCM [33]. Oxidative stress is provoked when ROS levels increase and are not compensated by endogenous antioxidant systems such as SOD [34]. Excessive instability of Fe^2+^ increases the risks of oxidative stress-evoked injury [35]. MDA is one of the most commonly evaluated markers of oxidative stress [36]. Our results demonstrate that after DEX treatment, HG-induced cardiomyocytes exhibited decreased MDA and ROS levels and Fe^2+^ concentration, as well as increased SOD activity. Previous studies have shown that DEX can reduce oxidative stress and H9C2 cell necroptosis and apoptosis [37, 38]. Furthermore, DEX has been shown to attenuate cardiomyocyte ferroptosis in myocardial I/R injury [13, 23]. These findings suggest that DEX may exert anti-ferroptotic effects on cardiomyocytes in DCM.

Recent studies indicate that the post-translational modification of GPX4 could be a potential target for treating ferroptosis-associated conditions [12]. Nrf2, as a transcription factor, plays a critical role in regulating ferroptosis [39]. Herein, we uncovered that Nrf2 nuclear levels and GPX4 levels were diminished in HG-induced cardiomyocytes. However, DEX treatment has been shown to increase Nrf2 nuclear translocation in H9C2 cells exposed to cobalt chloride [40]. In addition, DEX has been shown to elevate the levels of GPX4 and nuclear Nrf2 in HG-induced cardiomyocytes. Moreover, a prior study indicated that DEX could significantly augment the levels of Nrf2 and GPX4 in cardiomyocytes exposed to hypoxia/reoxygenation [13]. These findings suggest that DEX may activate the Nrf2/GPX4 pathway by facilitating Nrf2 nuclear translocation. Nrf2 has been demonstrated to protect cardiac cells and the heart from high glucose-induced injury in vitro [41]. Interestingly, Nrf2 can modulate the levels of oxidant signaling protein levels, which play a crucial role in programmed cellular functions [42]. Inhibition of Nrf2 using the specific inhibitor ML385 resulted in reduced cardiomyocyte viability, increased apoptosis, downregulated Bcl2 and SOD, and upregulated Bax, Fe^2+^, MDA, and ROS. The activation of the Nrf2/ferroportin1 pathway has been shown to mitigate myocardial I/R injury in diabetic rats by modulating ferroptosis and iron homeostasis [43]. Moreover, Nrf2 deficiency exacerbates cardiac injury induced by Angiotensin II [44]. Transcriptional activation of Nrf2 has been found to be protective against ferroptosis, while Nrf2 inhibition averts resistance to GPX4 inhibitor-induced ferroptosis in head and neck cancer [45]. These findings suggest that DEX up-regulates GPX4 and represses ferroptosis by increasing Nrf2 nuclear translocation, thus alleviating HG-induced cardiomyocyte injury. However, Nrf2 inhibition could partly negate the protective effect of DEX on HG-induced cardiomyocytes.

Overall, the current study suggests that DEX has the potential to alleviate HG-induced cardiomyocyte injury by upregulating GPX4 and increasing the nuclear translocation of Nrf2, thus inhibiting ferroptosis. However, there are still limitations to this study, such as the lack of immunofluorescence validation for Nrf2 expression through immunofluorescence and the absence of animal experiments. Further research is needed to determine the optimal dosage of DEX and explore other regulatory mechanisms involved in DCM. Nonetheless, the findings of this study provide insights into the potential therapeutic effects of DEX in the treatment of DCM.

## Data Availability

The data that support the findings of this study are available from the corresponding author upon reasonable request.
